# Hydrogels for Peripheral Nerve Repair: Emerging Materials and Therapeutic Applications

**DOI:** 10.3390/gels11020126

**Published:** 2025-02-09

**Authors:** Oana Taisescu, Venera Cristina Dinescu, Alexandra Daniela Rotaru-Zavaleanu, Andrei Gresita, Michael Hadjiargyrou

**Affiliations:** 1 Department of Human Anatomy, University of Medicine and Pharmacy of Craiova, 2–4 Petru Rares Str., 200349 Craiova, Romania; oana.taisescu@umfcv.ro; 2 Department of Health Promotion and Occupational Medicine, University of Medicine and Pharmacy of Craiova, 2–4 Petru Rares Str., 200349 Craiova, Romania; 3 Department of Epidemiology, University of Medicine and Pharmacy of Craiova, 2–4 Petru Rares Str., 200349 Craiova, Romania; alexandra.rotaru@umfcv.ro; 4 Experimental Research Centre for Normal and Pathological Aging, University of Medicine and Pharmacy of Craiova, 200349 Craiova, Romania; andrei.gresita@umfcv.ro; 5 Department of Physiology, University of Medicine and Pharmacy of Craiova, 2–4 Petru Rares Str., 200349 Craiova, Romania; 6 Department of Biological & Chemical Sciences, New York Institute of Technology, Old Westbury, NY 11568, USA

**Keywords:** peripheral nerve injury, functionalized hydrogels, nerve regeneration, tissue engineering, bioactive molecules, axonal growth, neuronal survival, inflammation modulation

## Abstract

Peripheral nerve injuries pose a significant clinical challenge due to the complex biological processes involved in nerve repair and their limited regenerative capacity. Despite advances in surgical techniques, conventional treatments, such as nerve autografts, are faced with limitations like donor site morbidity and inconsistent functional outcomes. As such, there is a growing interest in new, novel, and innovative strategies to enhance nerve regeneration. Tissue engineering/regenerative medicine and its use of biomaterials is an emerging example of an innovative strategy. Within the realm of tissue engineering, functionalized hydrogels have gained considerable attention due to their ability to mimic the extracellular matrix, support cell growth and differentiation, and even deliver bioactive molecules that can promote nerve repair. These hydrogels can be engineered to incorporate growth factors, bioactive peptides, and stem cells, creating a conducive microenvironment for cellular growth and axonal regeneration. Recent advancements in materials as well as cell biology have led to the development of sophisticated hydrogel systems, that not only provide structural support, but also actively modulate inflammation, promote cell recruitment, and stimulate neurogenesis. This review explores the potential of functionalized hydrogels for peripheral nerve repair, highlighting their composition, biofunctionalization, and mechanisms of action. A comprehensive analysis of preclinical studies provides insights into the efficacy of these hydrogels in promoting axonal growth, neuronal survival, nerve regeneration, and, ultimately, functional recovery. Thus, this review aims to illuminate the promise of functionalized hydrogels as a transformative tool in the field of peripheral nerve regeneration, bridging the gap between biological complexity and clinical feasibility.

## 1. Background

Peripheral nerve injuries continue to represent a formidable clinical challenge, largely due to the intricate biological processes required for effective nerve repair and the inherently limited regenerative capacity of the peripheral nervous system [1]. Such injuries often lead to profound sensory and motor deficits, severely compromising the quality of life of affected individuals, and have significant socioeconomic burdens [2]. Despite advances in microsurgical techniques, current gold-standard treatments, such as nerve autografts, face significant limitations such as donor site morbidity, finite donor tissue availability, and inconsistent functional outcomes, particularly in cases involving extensive nerve defects or delayed intervention [3]. Consequently, there is a growing interest in exploring innovative and integrative strategies aimed at enhancing nerve regeneration. Among these, tissue engineering via biomaterial-based approaches has garnered significant attention and offers promising solutions to address the shortcomings of conventional therapies by providing a more favorable microenvironment conducive for nerve regeneration and functional restoration [4].

Tissue engineering approaches utilize biomaterials to create supportive scaffolds that mimic the native extracellular matrix (ECM), guiding axonal growth and providing essential structural and biochemical cues for nerve regeneration [5]. Hydrogels, in particular, have garnered significant attention due to their unique physicochemical properties, including biocompatibility, tunable mechanical properties, and the ability to support cell attachment, proliferation, and differentiation [6]. Additionally, these versatile biomaterials can be engineered to incorporate a variety of bioactive molecules and growth factors, facilitating controlled release and creating a microenvironment conducive to nerve repair [7]. For instance, bioactive molecules such as laminin-derived peptides (e.g., IKVAV and YIGSR) are frequently used to promote neurite outgrowth and cell adhesion [8]. Growth factors such as nerve growth factor (NGF), brain-derived neurotrophic factor (BDNF), and glial cell line-derived neurotrophic factor (GDNF) have also been integrated into hydrogels to enhance neuronal survival and axonal growth [9]. Further, hydrogels can also be seeded with Schwann cells, crucial for myelination and axonal guidance, or mesenchymal stem cells (MSCs), which can secrete trophic factors and modulate the inflammatory response, thus creating a regenerative niche tailored for peripheral nerve repair [10]. Recent advancements in hydrogel technology have also enabled the development of sophisticated systems that not only provide structural support but also actively promote cellular recruitment, angiogenesis, and neurogenesis [11]. These innovations have emerged as one of the most promising tools in the field of peripheral nerve repair, bridging the gap between biological complexity and clinical feasibility [12].

Despite this progress, a comprehensive synthesis of existing research that addresses new materials, their therapeutic applications, and the associated translational challenges remains noticeably absent. This review provides a synthesis of the latest knowledge concerning the design, functionality, and therapeutic efficacy of hydrogels in peripheral nerve injuries. It aims to serve as an inclusive and cohesive study that addresses the bioengineering principles underlying hydrogel applications along with their preclinical validation. Further, this review critically examines the translatability of preclinical research findings into the clinic. As such, it not only highlights the necessity of advancing the clinical relevance of hydrogel-based therapies but also recognizes the need for new experimental designs and new translational strategies. By addressing these crucial aspects, this review aims to provide insights for researchers, clinicians, and biomaterials scientists who are actively involved in this dynamic and exciting field.

### 1.1. Classification of Peripheric Nerve Injury

**Figure 1:**
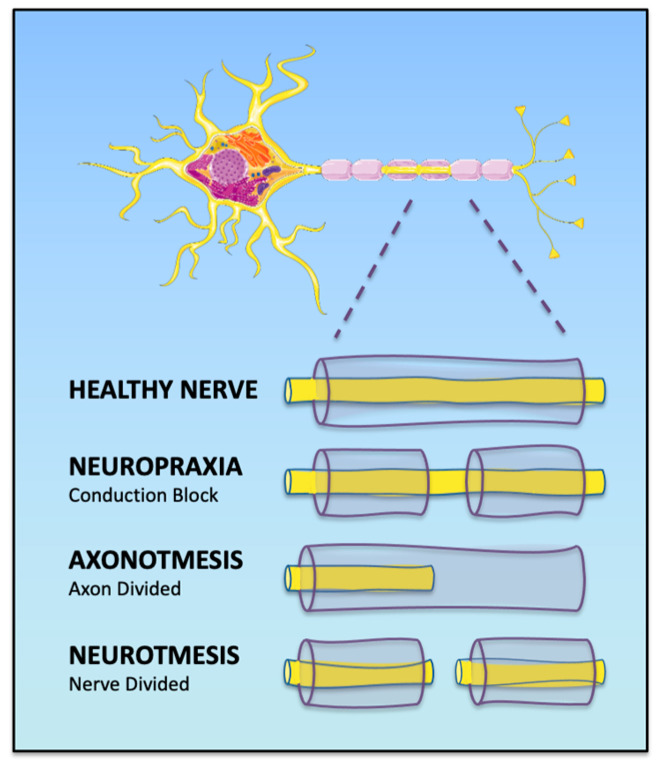
Schematic representation of Seddon’s classification of peripheral nerve injury. This figure was generated using Servier Medical Art. The selected artwork (cell shown in the figure) was taken or adapted from pictures provided by Servier Medical Art (Servier; https://smart.servier.com/, accessed on 15 January 2025), licensed under a Creative Commons Attribution 4.0 Unported License [14].

Peripheral nerve injuries are classified based on the severity and extent of damage to nerve structures, with three primary types: neuropraxia, axonotmesis, and neurotmesis [1] (Figure 1). Autografts, considered the gold standard for bridging nerve gaps, involve transplanting a donor nerve to the injury site, but have significant limitations, such as donor site morbidity, limited graft availability, mismatch in diameters between donor and recipient nerves, and potential for neuroma formation [13]. As such, they are not ideal for peripheral nerve regeneration and thus the need for more sophisticated approaches.

### 1.2. Common Pathologies and Occupational Implications

Peripheral nerve injuries represent a significant clinical and occupational health issue, often resulting in long-term disability and substantial socioeconomic impact [15]. These injuries commonly occur as a result of trauma, repetitive strain, or compression and are frequently associated with motor vehicle accidents, falls, and penetrating injuries (i.e., knife and glass wounds) [16]. From an occupational perspective, individuals involved in physically demanding jobs, such as construction workers, assembly line operators, and healthcare professionals, are at higher risk of developing nerve lesions due to repetitive movements, heavy lifting, or sustained pressure on specific nerve pathways [17]. The clinical manifestations of peripheral nerve injuries depend on the location and extent of the damage [2]. Common symptoms include weakness, loss of sensation, and impaired motor function, which can significantly impact a patients’ quality of life. In occupations requiring fine motor skills or heavy manual labor, such impairments often lead to loss of employment and/or a need for prolonged rehabilitation [18]. For instance, repetitive strain injuries in factory workers frequently lead to conditions such as carpal tunnel syndrome, a compression of the median nerve at the wrist [19]. Similarly, ulnar nerve entrapment, prevalent among manual laborers and athletes, can result from prolonged pressure on the elbow or forearm [20].

### 1.3. Therapeutic Strategies

Current therapeutic strategies aim to restore nerve continuity, promote axonal regeneration, and recover functional outcomes [21]. Surgical intervention remains the gold standard of treatment for severe nerve lesions, particularly those involving transection or extensive damage [1]. Direct end-to-end repair is ideal for clean cuts with minimal nerve tissue loss [22]. However, when a tension-free repair is not feasible, nerve grafting is employed to bridge the gap [23]. Autografts, harvested from donor nerves like the sural nerve, are the standard approach, although their use is limited by donor site morbidity and restricted availability [24]. Nerve allografts offer an alternative for larger defects, but their application is often constrained by the need for immunosuppression, thereby complicating the patient’s life [25].

In recent years, advances in biomaterials have ushered new possibilities for peripheral nerve repair. Synthetic and biological nerve conduits, as well as hydrogels, are being developed to create scaffolds that support axonal growth and improve regeneration [26]. Functionalized hydrogels, in particular, show significant potential by mimicking the tissue’s natural ECM, delivering bioactive molecules, and modulating inflammation to enhance recovery [27]. Hydrogels offer tunable mechanical properties, biocompatibility, and capacity for biofunctionalization, making them promising candidates for addressing the unmet therapeutic needs for nerve regeneration [28]. This review examines preclinical evidence to assess the efficacy and mechanisms of functionalized hydrogels in neural repair.

## 2. Material and Methods

**Figure 2:**
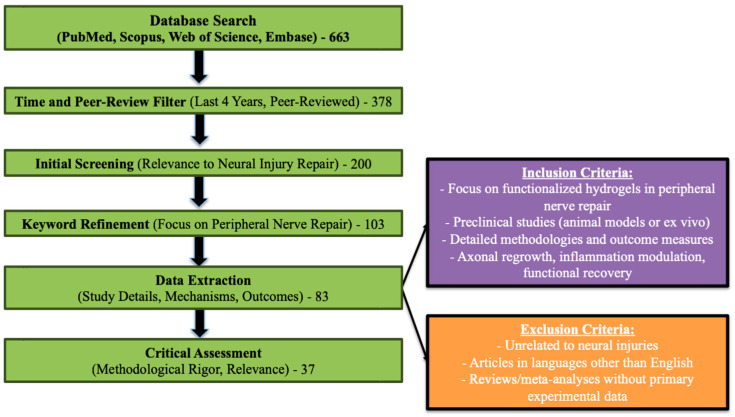
Workflow chart for selecting and assessing functionalized hydrogel studies in peripheral nerve repair.

For this review, a comprehensive literature search was conducted across PubMed, Scopus, Web of Science, and Embase to identify recent advancements in peripheral nerve repair using functionalized hydrogels. Keywords such as “functionalized hydrogels”, “neural injury repair”, “peripheral nerve regeneration”, and “biofunctionalized hydrogels” were employed. The search was restricted to peer-reviewed journal articles published within the last four years to ensure current relevance. The inclusion criteria targeted studies investigating functionalized hydrogels in peripheral nerve repair, with a focus on preclinical models (animal or ex vivo). Eligible studies provided detailed methodologies, including hydrogel composition, functionalization techniques, and effects on neural repair. Outcome measures such as axonal regrowth, inflammation modulation, or functional recovery were prioritized. Exclusion criteria filtered out studies unrelated to neural injuries, non-English publications, and reviews or meta-analyses lacking primary data. Key data were extracted from the selected studies, including publication details, study design, hydrogel types, functionalization strategies, mechanisms of action, and outcomes related to nerve regeneration. Specific emphasis was placed on the impact of hydrogel functionalization on axonal growth, neuronal survival, immune modulation, and scaffold integration within the injury site. The methodological rigor of the included studies was critically assessed (Figure 2).

## 3. Mechanisms of Action in Peripheral Nerve Regeneration

Hydrogels facilitate peripheral nerve regeneration via their intricate molecular interactions with neural and inflammatory pathways [29]. Specifically, these biomaterials serve as platforms for the delivery of growth factors, drugs, and cells, guiding axonal growth, and modulating inflammation [30]. Their molecular structure and functionalization directly influence critical signaling cascades that drive nerve repair and regeneration [27]. As delivery systems, hydrogels facilitate the controlled release of growth factors such as NGF, BDNF, and GDNF [31]. In turn, these factors activate key signaling pathways, including the phosphoinositide 3-kinase (PI3K)/Akt pathway, which promotes neuronal survival and axonal elongation, and the Ras/ERK pathway, which regulates cellular differentiation and neurite outgrowth [9]. The incorporation of growth factors into hydrogels ensures sustained bioavailability, preventing the rapid degradation typically associated with free-floating proteins [32]. Functionalized hydrogels with heparin or other glycosaminoglycans further enhance the binding and stabilization of growth factors, ensuring their interaction with high-affinity receptors on native neurons and Schwann cells [33].

Hydrogels can also act as scaffolds that guide axonal regeneration by providing physical cues that align axons and biochemical signals that activate cellular pathways [34]. The mechanical properties of hydrogels, such as stiffness and elasticity, are carefully tuned to mimic the ECM, thereby engaging integrin-mediated signaling pathways [35]. Integrins, transmembrane receptors that link the ECM to the cytoskeleton, trigger focal adhesion kinase (FAK) activation upon interaction [36]. This activation promotes cytoskeletal reorganization and axonal migration, which are critical for nerve regrowth [36]. Additionally, conductive hydrogels, incorporating materials like polypyrene or graphene, facilitate electrical stimulation, which activates voltage-gated calcium channels [37]. The subsequent calcium influx induces the activation of CaMKII and CREB, further promoting neurite outgrowth and even synaptic plasticity [38].

Hydrogels can also be designed to target pathways that regulate immune cell activity and cytokine release, and this plays a role in inflammation modulation [39]. Following peripheral nerve injury, the activation of Toll-like receptors (TLRs) on macrophages and Schwann cells triggers the NF-κB signaling pathway, resulting in the production of pro-inflammatory cytokines such as TNF-α and IL-6 [40]. Hydrogels loaded with anti-inflammatory agents, corticosteroids, or non-steroidal anti-inflammatory drugs (NSAIDs) inhibit this cascade by blocking NF-κB activation, reducing the inflammatory response and preventing tissue damage [41]. Some hydrogels are also designed to release molecules such as IL-10 or TGF-β, which activate anti-inflammatory pathways and promote the transition of macrophages from a pro-inflammatory (M1) to a pro-regenerative (M2) phenotype. This phenotypic switch is essential for creating an environment conducive to nerve repair and axonal regrowth [42]. Further, hydrogels can encapsulate antioxidants such as N-acetylcysteine (NAC) or superoxide dismutase (SOD), which reduce oxidative stress by scavenging reactive oxygen species (ROS) [43]. Excessive ROS production, a hallmark of inflammation following nerve injury, disrupts cellular homeostasis and exacerbates neuronal apoptosis. By neutralizing ROS, hydrogels help maintain the integrity of neuronal mitochondria and reduce the activation of apoptosis-related pathways such as JNK and caspase cascades [44]. Neuroprotection is further enhanced by the delivery of neuroprotective agents that modulate pathways like PI3K/Akt and MAPK, ensuring neuronal survival and functional recovery [45]. In addition to modulating inflammation, providing neuroprotection, and promoting macrophage polarization toward a regenerative phenotype [46], hydrogels can play a critical role in tissue reconstruction and vascularization by incorporating angiogenic factors that stimulate angiogenesis [47]. Depending on their design, biofunctionalized hydrogels can integrate peptides, ECM components, or ligands that enhance cell attachment, migration, and tissue integration. Moreover, stimuli-responsive hydrogels have the capacity to release therapeutic agents in response to environmental triggers such as pH, temperature, or electrical signals [48]. Conductive hydrogels, modified with polymers like polypyrrole or graphene, facilitate electrical signal transmission, further improving nerve repair outcomes [49]. These materials can be classified into natural hydrogels (e.g., alginate, collagen, chitosan), known for their superior biocompatibility; synthetic hydrogels (e.g., PEG, PVA), which offer tunable mechanical properties; and hybrid hydrogels that combine natural and synthetic elements to optimize biofunctionality. The versatility of these materials extends across a wide range of applications, from promoting regeneration in peripheral nerves and spinal cord injuries to advancing treatments for central nervous system disorders, thereby highlighting their broad potential in regenerative medicine and neurology [50].

### 3.1. Hydrogel-Based Conduits for Peripheral Nerve Repair: Advantages, Emerging Materials, Mechanical Properties, and Fabrication Techniques

#### 3.1.1. Advantages of Hydrogel-Based Conduits in Peripheral Nerve Repair

Hydrogel-based conduits, with their high water content and viscoelastic properties, provide a biomimetic environment that closely resembles the natural ECM, which is crucial for supporting cell growth fundamental to nerve regeneration [27]. This gel-like nature facilitates enhanced cell adhesion, proliferation, and differentiation [51]. These characteristics are less pronounced in more rigid scaffolds such as thermoplastic conduits. While thermoplastic materials provide robust mechanical support, they often lack bioactivity and cell signaling capabilities intrinsic to hydrogels, making them more useful in applications where rigidity and long-term stability are required [52]. For example, in prior research conducted by our group, we demonstrated that the efficacy of seeding hydroxyapatite scaffolds with osteoblastic cells was significantly enhanced by coating the scaffold surfaces with collagen; this modification improved cell adhesion and proliferation, underscoring the importance of surface biofunctionalization in scaffold design [53]. Additional research suggests that seeding scaffolds with cells could create a natural network that would in turn integrate within existing tissues [54]. Hydrogels are inherently versatile in their ability to be functionalized with bioactive molecules, including peptides, small molecules, or growth factors. This functionalization can be utilized to control the release kinetics of these molecules, thereby sustaining the bioactive cues essential for prolonged regenerative processes [55]. Such controlled release is less feasible in nanofibers and thermoplastics, where the incorporation and sustained delivery of bioactive substances pose significant challenges and might require additional coatings with biomimicking materials [56].

Notably, the elasticity and injectability of certain hydrogels offers a practical advantage; allowing for the scaffolds to be introduced to the injury site via minimally invasive procedures. This capability enables them to conform to the complex anatomical geometries of nerve injury sites, a significant advantage over pre-formed rigid conduits, which cannot adapt to irregular defects without considerable manipulation and potential traumatic intervention [57]. Moreover, the risk of damaging rigid biocompatible structures while implanting is high, and it is worth noting that nerves often require high flexibility; thus, rigid structural recovery could compromise nerve mobility and functionality [58].

In summary, while nanofibers and thermoplastic conduits have their own advantages in terms of structural integrity and, at times, simpler manufacturing processes, hydrogel-based conduits offer superior bioactivity, elasticity, customization of mechanical and biochemical properties, and application versatility, making them particularly suitable for the complex environment and process of nerve repair [59].

#### 3.1.2. Emerging Materials and Mechanical Properties

As nerve damage extent and location can vary, the need for highly personalized hydrogel conduits is high. An ideal nerve conduit for peripheral nerve repair must meet several criteria, including biological, mechanical, and functional requirements, to effectively support nerve regeneration. Biologically, the conduit should be biocompatible, non-toxic, and capable of promoting cell adhesion, proliferation, and differentiation [26]. It should degrade at a rate that matches tissue regeneration, leaving no harmful by-products, mimicking the ECM to guide axonal growth while preventing scar tissue formation [60]. Mechanically, the conduit should have sufficient tensile and compressive strength to maintain structural integrity and elasticity to accommodate movement, and should be porous, with an optimal pore size for nutrient diffusion and cell infiltration [61]. Functionally, the design should provide channeled guidance for directional axonal growth and vascularization, and potentially incorporate bioactive molecules or electrical conductivity to enhance nerve repair [58].

The mechanical attributes of hydrogels, such as compressive strength, elasticity, and viscoelasticity, are critical for providing structural support while accommodating the dynamic environment of peripheral nerves. Advances in materials and fabrication techniques, have made it possible to develop conduits with tunable properties to meet these requirements. A diverse array of materials makes hydrogel design extremely versatile and could in turn be translated into highly personalized applications to address individual disabilities. Mechanical factors could play a fundamental role in tailoring hydrogels for precise lesion damage, size, and long-term durability. In a recent study, Liu et al. (2022) developed a fibrin hydrogel with optimized elasticity and porosity, ensuring compressive strength and promoting nutrient diffusion [62]. Similarly, Zhang et al. (2023) designed chitin-based conduits combined with growth factor-loaded hydrogels, where the viscoelastic properties aligned with nerve tissue mechanics, facilitating prolonged therapeutic effects [63]. Shen et al. (2022) introduced chitosan conduits with peptide nanofiber scaffolds exhibiting appropriate stiffness to support cell adhesion and axonal growth, while Meder et al. (2021) developed a nerve-specific ECM hydrogel derived from decellularized porcine nerve tissue, closely mimicking native neural environments with optimal viscoelastic properties [64,65]. These studies highlight the importance of tailoring hydrogel stiffness and degradation rates to support nerve regeneration while minimizing mechanical mismatch with native tissues. Lastly, computational approaches can also be employed to model scaffold elasticity and porosity [66].

Recent research has also introduced novel hydrogel formulations enriched with bioactive components to enhance the regenerative potential. Self-assembly peptides, as demonstrated by Lopez-Silva et al. (2021) and Yang et al. (2020), formed nanofibrous hydrogels that mimicked the ECM and promoted cell infiltration, Schwann cell activity, and axonal regeneration [67,68]. Magnesium-enriched hydrogels were developed by Gao et al. (2024) and Yao et al. (2022), which utilized magnesium nanoparticles to support neuronal function through ionic signaling and accelerated axonal regrowth [69,70]. Bioinspired polymers, such as the self-healing conductive hydrogel developed by Xuan et al. (2023) based on hyaluronic acid and cystamine, enhanced Schwann cell myelination and functional recovery [71]. Additionally, decellularized ECM hydrogels were utilized by Gregory et al. (2022) and Kellaway et al. (2023), showcasing their ability to retain critical ECM proteins like laminin and collagen, providing tailored biochemical signals and structural properties to address specific challenges in peripheral nerve repair [72,73].

#### 3.1.3. Fabrication Techniques, Compatibility, and Evaluation

The fabrication techniques used to produce hydrogel conduits have advanced significantly. The development of these sophisticated scaffolds incorporates state-of-the-art fabrication technologies such as electrospinning, 3D printing, and conductive material integration. For instance, Zheng et al. (2021) employed electrospinning to create aligned nanofiber scaffolds integrated with decellularized matrix hydrogels, promoting directional axonal growth [74]. This technique allows for the creation of nanofibrous scaffolds that mimic the ECM and could in turn direct axonal growth due to their aligned geometry. On the other hand, 3D printing offers control over the scaffold’s porosity and geometry, enabling customization to specific injury topography. This technique also allows for the integration of bioactive compounds directly into the scaffold [75]. Lee et al. (2022) used 3D printing to fabricate porous PLCL conduits with hydrogel-based microgrooved surfaces, guiding Schwann cell alignment and axonal regrowth [76]. Additionally, Liu et al. (2021) developed a self-curling electroconductive nerve dressing that ensured close contact with the nerve, particularly for diabetic neuropathy treatment [77]. The inclusion of conductive materials like graphene or polypyrrole enhances the electrical properties of the hydrogels, crucial for supporting nerve tissue electrical signaling and regeneration [78,79]. These innovative fabrication approaches allow precise customization and fine tuning of scaffold architecture, enabling better integration with host tissues and localized therapeutic delivery. The in vitro applicability of these structures has been evaluated in in vitro studies, such as those by Lopez-Silva et al. (2021) and Sun et al. (2024), which focused on promoting Schwann cell activity through improved adhesion, proliferation, and differentiation [67,80]. Furthermore, in vivo models, such as sciatic nerve defect models used by Liu et al. (2022) and Meder et al. (2021), demonstrate improved functional recovery, axonal growth, and myelination [62,65].

Importantly, the translational value of preclinical studies is directly influenced by the accuracy of the evaluation techniques being used. In order to precisely investigate the effectiveness of hydrogel-based conduits, functional and electrophysiological assessments were employed. As such, electrophysiological tests such as nerve conduction velocity (NCV) and electromyography (EMG) are fundamental for quantifying the restoration of nerve function, providing direct measurements of electrical signal transmission [19]. Further, behavioral tests are also crucial as they assess the functional recovery in animal models. Tests like the Basso, Beattie, and Bresnahan (BBB) scale [81], which measures motor recovery; CatWalk Gait Analysis, for detailed gait parameter analysis; the Rotarod Test, used to assess motor coordination and balance; and the Grid Walk Test, which evaluates motor coordination and limb placement accuracy, are all rigorously used [82]. Additionally, the Hindlimb Suspension Test has been proven to be important for assessing muscle strength and neuromuscular function, the Von Frey Filament Test has been used to assess sensory function and nociceptive thresholds, and the Hot Plate Test has been used to evaluate sensory and pain thresholds [83].

Histological analyses also complement these studies by examining tissue sections, especially for axonal regeneration and myelination [84]. Additionally, biocompatibility and toxicity tests ensure that the hydrogels do not induce adverse immune responses or systemic toxicity, affirming the safety of these materials for future clinical applications [85]. Nevertheless, the use of artificial intelligence and machine learning could further improve the accuracy of evaluating functional recovery. Together, these evaluation methods provide a comprehensive assessment of hydrogel structures, ensuring their safety as well as translation value in preclinical trials.

## 4. Applications of Hydrogels in Peripheral Nerve Repair

**Figure 3:**
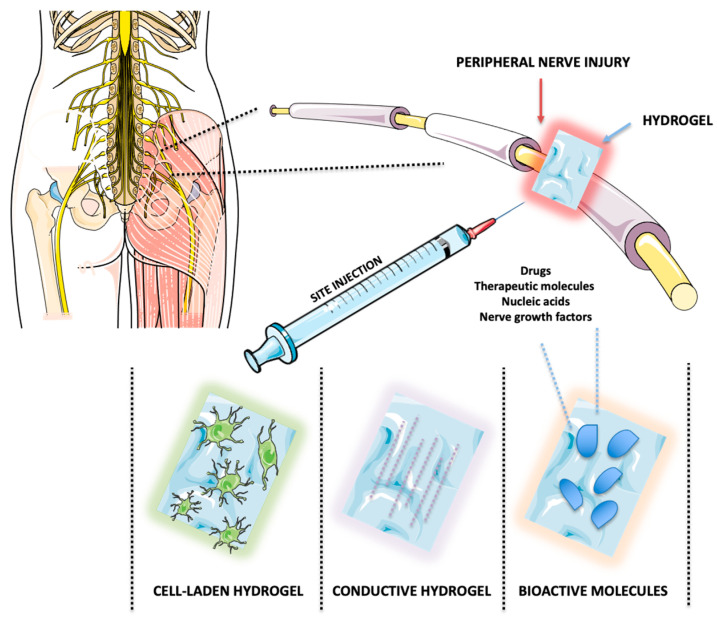
Illustration of hydrogel types used in peripheral nerve injury repair. This figure was generated using Servier Medical Art. Selected artwork (cells shown in the figure) was taken or adapted from pictures provided by Servier Medical Art (Servier; https://smart.servier.com/, accessed on 15 January 2025), licensed under a Creative Commons Attribution 4.0 Unported License [11].

As aforementioned, functionalized hydrogels provide diverse applications through various mechanisms of action and material compositions [27] by delivering therapeutics such as growth factors, drugs, and stem cells, providing a scaffold that supports axonal regeneration by closely mimicking the native ECM, or serving conductive purposes [6]. The following section delves into the specialized types of hydrogels designed for peripheral nerve regeneration, exploring their unique material compositions, bioactive integrations, and the sophisticated mechanisms characterizing their therapeutic efficacy (Figure 3).

### 4.1. Guiding Axonal Growth: Scaffold Hydrogels

Hydrogels can be tailored to address specific aspects of the regeneration process. For example, scaffold hydrogels are designed to direct axonal growth by providing both physical and biochemical cues. Functionalized with bioactive molecules such as laminin peptides, nerve-specific growth factors, or ECM components, these hydrogels can mimic the natural microenvironment necessary for axonal growth and alignment [86]. Additionally, they can create pathways for directed axonal regeneration, support Schwann cell adhesion, migration, and myelination, and incorporate biofunctional components to enhance neural cell interactions. Using a variety of innovative hydrogel materials, researchers have made remarkable progress in advancing nerve repair in preclinical models. Together, these studies form a coherent narrative of innovation and optimization in peripheral nerve regeneration (Table 1).

A recent study by Nawrotek et al. utilized polycaprolactone/chitosan–hydroxyapatite hydrogels and demonstrated their ability to enhance nerve guidance, axonal regrowth, and implant stability in vitro, highlighting their potential for peripheral nerve regeneration [87]. Another investigation employing fibrin hydrogels incorporated with Wnt5a revealed improved axonal growth, Schwann cell proliferation, and the secretion of VEGF and NGF, key factors essential for creating a regenerative microenvironment in vivo [62]. Similarly, research combining VEGF and NGF with gelatin methacrylate hydrogels and chitin conduits showed significant improvements in nerve conduction velocity and muscle preservation, underscoring the efficacy of bioactive molecule integration in promoting functional recovery [63]. Furthermore, a study by Lopez-Silva et al. (2021) on the use of multidomain peptide hydrogels demonstrated their capacity to recruit macrophages for debris clearance and enhance remyelination, effectively accelerating nerve function restoration in rats [67].

Further advancements in hydrogel design have emphasized structural and biochemical precision. For instance, a study by Shen et al. (2022) demonstrated that self-assembling peptide nanofiber scaffolds supported nerve repair and muscle innervation in rats, underscoring the potential of integrating bioactive peptides with structural conduits [64]. Similarly, research on decellularized ECM hydrogels revealed their ability to provide a robust structural scaffold while aligning Schwann cells and promoting vascularization, showcasing the synergistic potential of bio-derived materials with cellular regenerative mechanisms [72]. Meder et al. (2021) increased our understanding of ECM hydrogels by optimizing formulations derived from porcine nerves. Their work connected structural integrity with functional outcomes, such as axon count and gait recovery, underscoring the importance of material optimization for long-term efficacy [65]. Similarly, a study by Yang et al. (2022) explored peptide hydrogels with RAD-based motifs, showing how minimal variations in peptide composition can influence Schwann cell adhesion, axonal regeneration, and functional recovery [88]. Lee et al. (2022) expanded the scope of hydrogel functionality by integrating poly(lactide-co-ε-caprolactone) (PLCL) and gelatin into a light-crosslinked system, emphasizing how material properties like elasticity and crosslinking can influence cell adhesion and axonal regeneration [76]. Notably, cell integration and synaptic activity within existing neural networks are critical for successful nerve repair, making the incorporation of conductive materials an essential strategy. For example, Amagat and colleagues enhanced both electrical conductivity and anisotropic guidance, two pivotal factors for promoting precise axonal growth, by integrating graphitic carbon nitride and reduced graphene oxide into hydrogel scaffolds [89]. Collectively, these innovative biomaterials not only supported the establishment of functional synapses but also facilitated the alignment and targeted regeneration of neural pathways, underscoring their transformative potential in neural tissue engineering.

The integration of bioactive and structural properties was also utilized in a recent study that combined GelMA and SF-MA 3D-printed hydrogels to create regenerative microenvironments. This work demonstrated the importance of scaffolds that not only support cellular adhesion and migration but also facilitate myelination and functional recovery [90]. Takeya et al. (2023) further exemplified the dual-purpose design of hydrogels by encapsulating Schwann cells within chitosan–collagen conduits, providing both cellular and structural support [91]. This strategy directly followed the findings of Kuna et al. (2022), whose nerve-derived hydrogels created a supportive niche for sensory neurons and Schwann cells, driving axonal regrowth and functional recovery [92]. Lastly, a recent study by Zheng et al. (2023) utilized polydopamine nanoparticles/hyaluronic acid methacryloyl hydrogel-based scaffolds and achieved outcomes comparable to autografts [74].

### 4.2. Reducing Scar Tissue: Barrier Hydrogels

Barrier hydrogels have emerged as a transformative solution to one of the most critical challenges in nerve repair: the inhibition of fibrotic scar formation (Table 2). Scar tissue, caused by fibroblast proliferation and excessive ECM deposition, acts as a physical and biochemical barrier to nerve regeneration. Barrier hydrogels tackle this issue by creating a physical shield that prevents fibroblast invasion while releasing anti-fibrotic or anti-inflammatory agents to modulate ECM deposition [27]. Further, these hydrogels foster a pro-regenerative environment by promoting macrophage polarization toward the M2 phenotype [46]. Yang et al. (2023) exemplified this approach by developing bionic peptide hydrogel scaffolds functionalized with M2-derived cytokines and extracellular vesicles and revealed enhanced Schwann cell migration and macrophage transformation, as well as significant axonal regeneration and improved functional recovery in a rat sciatic nerve gap model [93]. These findings emphasize the importance of combining biochemical signals with structural support to address long-distance peripheral nerve injuries [93]. Building on this principle, Xue et al. (2023) designed a dual-network nerve-adhesive hydrogel composed of dopamine–isothiocyanate-modified hyaluronic acid and decellularized nerve matrix and showed a dual advantage in both in vitro and in vivo models by promoting Schwann cell proliferation and axonal outgrowth while simultaneously reducing inflammation and fibrosis [94]. Also, in rat sciatic nerve transection models, these properties translated to better motor and sensory recovery and enhanced muscle contraction, highlighting the hydrogel’s ability to mitigate both physical and biochemical barriers to nerve repair [94].

Another innovative approach utilized ROS-triggered H2S-releasing thermosensitive poly(amino acid) hydrogel (mPEG-PA-PP). This system introduced a dynamic mechanism for addressing oxidative stress and inflammation, key impediments in transected nerve injuries. In vitro studies demonstrated enhanced Schwann cell function, macrophage modulation, and endothelial cell activity, while in vivo data showed reduced oxidative stress, increased angiogenesis, and improved motor skill recovery [95]. The ability of this hydrogel to simultaneously address multiple regeneration pathways underscores its versatility [95]. Notably, Zhan et al. (2023) targeted the challenge of nerve adhesion using a polydopamine nanoparticles and hyaluronic acid methacryloyl (PDA NPs and HAMA) hydrogel. In a rat sciatic nerve adhesion model, this hydrogel effectively reduced nerve adhesion, enhanced motor nerve conduction, and improved overall function. The hydrogel’s anti-inflammatory properties further contributed to a favorable regenerative microenvironment, making it a dual-purpose solution for mitigating scar tissue and promoting nerve repair [96]. Despite the promising advancements in barrier hydrogel technology, the number of studies investigating their application in reducing scar tissue and promoting nerve repair remains relatively limited. This scarcity can be attributed to several factors, including the complexity of designing such dual-function hydrogels that simultaneously target multiple biological processes.

### 4.3. Enhancing Electrical Conductivity: Conductive Hydrogels

Conductive hydrogels have emerged as a transformative solution for restoring electrical signal transmission across nerve gaps, a critical requirement for functional recovery [27]. Enhanced with conductive polymers such as polypyrrole or graphene oxide, these materials facilitate electrical stimulation and activate neurons and Schwann cells, improve electrophysiological connections, and provide a biocompatible scaffold for nerve regeneration (Table 3). This multifaceted functionality could make conductive hydrogels fundamental for advanced nerve repair applications [97].

A recent study by Liu et al. (2021) utilized an electroconductive hydrogel in diabetic sciatic nerve injury models and demonstrated significant improvements in axonal regeneration, remyelination, motor function, and muscle denervation atrophy. This approach not only addressed nerve damage but also tackled the systemic challenges associated with diabetes [77]. In another innovative study using a rat diabetic sciatic nerve injury model, a hydrogel incorporating polypyrrole (PPy) and tannic acid (TA), combined with ropivacaine microspheres (ECH-MS/ROP), provided long-acting analgesia while simultaneously enhancing axonal regeneration, myelination, and reducing muscle atrophy [98]. Another study explored the integration of a conductive electrolytic hydrogel within a silicone microfluidic nerve cuff for peripheral nerve injuries. This system enabled effective neuromodulation, reversible nerve blocking, and robust electrical stimulation, thus offering a promising alternative to traditional metal electrodes for neural interface approaches [99]. Similarly, Srinivasan et al. (2023) investigated an alginate/polyacrylamide hydrogel functionalized with gold nanoparticles and showed improved electrical conductivity with facilitated electrophysiological recovery and supported axonogenesis in both rodent and porcine models, leading to significant motor and sensory recovery, preservation of muscle mass, and atraumatic electrode removal [100]. A recent investigation appears to further advance this field by examining the synergistic effects of scaffold materials and electrical stimulation. By utilizing various hydrogel compositions such as collagen, alginate, GelMA, and PEGDA, Olguin et al. (2023) demonstrated enhanced PC12 cell differentiation and neurite outgrowth, underscoring the importance of combining scaffold biocompatibility with optimized electrical cues to guide axonal growth [101].

Innovations in optogenetics were explored by Liu et al. (2023) that developed fatigue-resistant polyvinyl alcohol (PVA) nanocrystalline hydrogel optical fibers. These hydrogels enabled stable optogenetic nerve modulation, reduced pain hypersensitivity, and facilitated motor recovery in mouse models, underscoring their potential to integrate electrical conductivity with novel therapeutic techniques [102]. Additionally, Xuan et al. (2023) developed a self-healing conductive hydrogel (HASPy) based on hyaluronic acid (HA), cystamine, and pyrrole-1-propionic acid and demonstrated enhanced Schwann cell proliferation, axonal regeneration, reduced inflammation, and improved remyelination in both in vitro and in vivo studies. Its self-healing properties further ensure durability and consistent performance in peripheral nerve repair [71]. While progress made in utilizing conductive hydrogels for nerve repair is promising, additional studies are needed to fully understand their long-term efficacy. The ability of these materials to restore electrical conductivity across a nerve gap is fundamental for facilitating cell integration within existing neural networks and supporting the functional recovery of damaged nerves.

### 4.4. Promoting Cellular Healing: Drug Delivery and Cell-Encapsulating Hydrogels

One of the most promising applications of hydrogels in tissue engineering/regenerative medicine is their role as drug delivery and cell-encapsulating systems. These hydrogels not only provide structural scaffolding but also enable the controlled, localized release of bioactive molecules and protect encapsulated cells from the damaging microenvironment at the injury site. These types of hydrogels have emerged as essential tools for overcoming the challenges associated with conventional drug delivery methods, such as systemic distribution, of target effects, and insufficient targeting of the injury site. These biomaterials are designed to mimic the ECM, thereby enhancing cell viability, integration, and function by providing a conducive environment for adhesion, proliferation, and, in some cases, differentiation of the encapsulated cells (Table 4). Hydrogels can incorporate and gradually release neurotrophic factors, or even phytotherapeutic agents [103]. Further, these hydrogels can act as vehicles for stem cells, facilitating their sustained release and promoting their differentiation and function at the injury site. This strategy is particularly important for bridging nerve gaps, as it ensures a continuous supply of regenerative cells and signals in a manner that is more controlled and less invasive than direct cell injection, essentially acting as cell reservoirs [85]. Notably, one of the significant challenges in nerve repair is the high incidence of cell death when cells are injected directly into the injury site or into blood stream, due to mechanical stresses, ischemia, or inflammation. Encapsulation of cells within hydrogels offers protection from these adverse conditions. Moreover, encapsulation also provides a spatially controlled release, preventing premature cell death. Consequently, these hydrogels not only mitigate the risks associated with direct injection but also improve the overall effectiveness of the regenerative process [85].

In a recent study, Sun et al. (2024) utilized curcumin-loaded keratin–chitosan hydrogels to enhance nerve regeneration using both in vitro Schwann cell cultures and in vivo rat sciatic nerve crush models. This approach significantly reduced inflammation, improved functional recovery, and mitigated muscle atrophy [80]. Similarly, the application of peripheral nerve matrix (PNM) hydrogels promoted Schwann cell migration, axonal outgrowth, and neuromuscular junction formation, leading to enhanced motor recovery and improved electrophysiological conduction following peripheral nerve transection [104]. Expanding the scope of hydrogel applications, decellularized peripheral nerve (iPN) hydrogels were investigated for their potential in both peripheral and central nervous system injuries. These hydrogels improved Schwann cell viability and supported astrocyte spreading in vitro, while preclinical injection models demonstrated their capability for combinatorial drug and cell therapy delivery [105]. Bioactive hydrogels, in combination with dental pulp stem cells, were also used to address large-gap peripheral nerve injuries and showed successful axonal growth and functional recovery while facilitating stem cell integration for improved nerve defect bridging [106].

Building upon existing advancements in hydrogel compositions, newer studies have explored a variety of innovative hydrogel formulations. For example, magnesium-enriched silk fibroin interpenetrating polymer network (IPN) hydrogels have demonstrated remarkable potential in facilitating axonal regeneration, myelination, and Schwann cell migration, leading to significant motor and sensory recovery in peripheral nerve injury models [69]. Similarly, thermosensitive hydrogels composed of pluronic–alginate–lysine–dextran were effective in promoting Schwann cell migration and axonal outgrowth, improving nerve conduction, and mitigating muscle atrophy [107]. In parallel, advances in hydrogel–cell interactions have led to the development of scaffolds that integrate neuronal stem cells, further enhancing regenerative potential. Laminin-modified gellan gum hydrogels, combined with NGF, were showed to significantly promote neuronal stem cell proliferation, differentiation, and neurogenesis while reducing apoptosis, offering a promising strategy for peripheral nerve injuries [108]. This approach complements the use of aligned fibrin/functionalized self-assembling peptide hydrogels, which supported Schwann cell alignment and neurotrophin secretion, ultimately aiding in axonal regeneration and functional recovery, making them analogous to autografts [68].

Moreover, efforts to modulate the cellular microenvironment within hydrogels have led to the development of scaffolds that combine sustained release of neurotrophic factors with immune modulation. Chitosan/polycaprolactone (CH/PCL) hydrogels functionalized with dopamine-modified NGF-loaded microspheres demonstrated sustained NGF release, promoted axonal growth, and activated monocytes to foster early nerve healing, highlighting the ability of these hydrogels to influence both biochemical and immune responses [109]. Additionally, magnesium-encapsulated bisphosphonate-based nanocomposite hydrogels also showed great promise in supporting axonal regeneration, remyelination, and muscle atrophy reduction, with substantial improvements in functional recovery [70].

Finally, the exploration of decellularized ECM (dECM) hydrogels derived from rat sciatic nerves offers a 3D culture environment that closely mimics physiological conditions, enhancing Schwann cell viability and function. These hydrogels not only facilitate cellular function but also serve as valuable platforms for investigating peripheral nerve diseases and injuries, bridging the gap between therapeutic applications and preclinical research [72]. Collectively, these advancements highlight the versatility of hydrogels in addressing the multifaceted challenges of peripheral nerve repair, offering promising solutions to enhance regeneration and ultimately, functional recovery.

**Table 4:** Drug delivery and cell-encapsulating hydrogels for promoting peripheral nerve repair.

Active Compounds/Features	Hydrogel Materials	Loaded Drugs/Bioactives	Study Model	Effects on Recovery	Ref.
Laminin and NGF	Laminin-modified gellan gum	NGF	In vitro and ex vivo	Enhanced neuronal proliferation, differentiation, reduced apoptosis	[108]
Extracellular vesicles (EVs)	Thermosensitive hydrogel (pluronic–alginate–lysine–dextran)	EVs from adipose-derived stem cells	In vitro and in vivo (rat model)	Promoted Schwann cell migration and proliferation, improved axonal outgrowth and nerve conduction	[109]
bFGF and DPSCs	Gelatin methacryloyl (GelMA)	bFGF and DPSCs	In vitro and in vivo (rat sciatic nerve)	Accelerated Schwann cell migration and functional recovery similar to autografts	[106]
Laminin, collagen	Peripheral nerve matrix (PNM) hydrogel	None	In vitro and in vivo	Enhanced axon extension and electrophysiological recovery, reduced muscle atrophy	[104]
Curcumin	Keratin–chitosan hydrogels	Curcumin	In vitro and in vivo (rat sciatic nerve)	Reduced inflammation, improved axonal regeneration and functional recovery	[80]
Magnesium nanoparticles	Silk fibroin IPN hydrogels	Magnesium nanoparticles	In vitro (SCs and macrophages)	Enhanced myelination, reduced muscle atrophy, improved sensory and motor function	[69]
Decellularized ECM	Decellularized peripheral nerve hydrogel	None	In vitro	Enhanced Schwann cell viability, improved structural and functional recovery	[105]
Functionalized peptides	Aligned fibrin/self-assembling peptide hydrogel	None	In vitro and in vivo	Promoted Schwann cell alignment, axonal regeneration, functional recovery comparable to autografts	[68]
NGF-loaded microspheres	Chitosan/polycaprolactone hydrogels	Dopamine-modified NGF	In vitro	Sustained NGF release, enhanced axonal growth, Schwann cell migration	[109]
Magnesium and bisphosphonates	Nanocomposite hydrogels	Magnesium and bisphosphonate	In vitro and in vivo (rat sciatic nerve)	Enhanced myelination and axonal regrowth, reduced inflammation and muscle atrophy	[70]
Collagen and laminin	Decellularized ECM hydrogels derived from nerves	None	In vitro	Improved neuronal differentiation and axonal guidance, retained key ECM proteins for structural support	[72]

## 5. Translational Barriers in Peripheral Nerve Repair

Peripheral nerve injuries continue to pose significant clinical challenges, with current treatments often failing to achieve complete functional recovery [110]. While substantial advancements have been made in biomaterials, cellular therapies, and surgical approaches, the translation of these innovations into clinical practice continues to encounter several obstacles [111]. Biological complexities such as the slow rate of axonal regeneration, the necessity for synchronized cellular processes like Schwann cell migration, and ECM remodeling remain significant barriers to effective recovery [112,113]. The inflammatory microenvironment that develops following nerve injury further complicates this process, particularly in conditions such as diabetes, where oxidative stress inhibits regeneration [113], or in the elderly population, where the regenerative response is not as robust. Technological limitations present another major challenge, as engineered hydrogels frequently lack the mechanical flexibility, biocompatibility, and scalability required for widespread clinical application [114]. Despite the successes observed in preclinical models, there are unresolved issues surrounding the controlled release of bioactive molecules and the long-term stability of these materials. In addition, regulatory and ethical concerns complicate the translation of these technologies into the clinic. Cell-based therapies, including stem cells or exosome-laden hydrogels, face rigorous safety and efficacy requirements, further delaying the approval process. Ethical concerns surrounding the use of embryonic stem cells or genetically engineered components also add another hurdle [115]. Moreover, socioeconomic barriers also restrict access to advanced nerve repair technologies, particularly in low-resource areas. High development costs, the need for specialized manufacturing, and the challenge of ensuring replicability on a large scale with minimal alterations limit the widespread implementation of these therapies. Notably, a recent search of clinicaltrials.gov reveals no current, ongoing, or previous trials using the terms “Peripheral Nerve Injury” and “hydrogels”. In contrast, a search on PubMed for the same terms returns 315 studies (with most in the last 6 years), emphasizing the growth in this field and the significant potential for continued research and development. Overcoming these challenges will require multidisciplinary efforts to optimize biomaterial design, enhance scalability, and foster collaborations between academia, industry, and regulatory agencies. However, by addressing these hurdles, the field of peripheral nerve repair can move closer to providing effective and accessible clinical therapies [27].

### FDA-Approved Materials

Currently, several FDA-approved nerve conduits are commercially available and show great promise for future therapeutic approaches. However, it is essential to also recognize the limitations associated with these materials which are crucial for further development and clinical applications. For example, NeuraGen^®^ and NeuroTube^®^, made from collagen and polyglycolic acid, respectively, represent typical FDA-approved products that, while biocompatible, face challenges such as potential inflammatory responses leading to scar tissue formation [116]. Moreover, these conduits often fail to effectively bridge long nerve gaps (over 3 cm), primarily due to their mechanical rigidity, which may not match the natural tissue, and their limited capacity for sustained delivery of growth factors, crucial for promoting nerve regeneration [117]. This mismatch and lack of functional adaptability highlight critical areas where next-generation conduits could provide significant improvements, particularly in handling complex and long-segment nerve injuries [118].

## 6. Discussion

The application of hydrogels in peripheral nerve repair represents a transformative approach, bridging the gap between biological complexity and clinical needs. These biomaterials have demonstrated significant preclinical potential in promoting axonal regeneration, remyelination, and functional recovery in numerous studies and showcased their ability to enhance nerve repair [119]. For instance, conductive hydrogels, by facilitating electrical signal transmission, play a critical role in restoring nerve function, as seen in studies where hydrogels incorporating polypyrrole or graphene oxide facilitated electrophysiological recovery and axonal outgrowth in nerve injury models [23]. These properties, along with the tunable mechanical characteristics, biocompatibility, and capacity for biofunctionalization, enable hydrogels to mimic the ECM, providing a supportive environment that fosters nerve regeneration and integration [120]. Further, hydrogel-based drug delivery systems have shown promise by enabling the controlled release of neurotrophic factors and therapeutic agents. For example, curcumin-loaded keratin–chitosan hydrogels significantly reduced inflammation and improved nerve regeneration in both in vitro and in vivo models [78].

However, despite these promising advancements, several challenges remain and hinder the direct translation of hydrogel-based therapies into clinical practice. One of the main issues is the variability in hydrogel formulations and preclinical study designs. Also, the inconsistency in experimental conditions further complicates the ability to draw definitive conclusions about their effectiveness. This in turn highlights the urgent need for standardized methodologies to enhance reproducibility and reliability [85]. Further, while hydrogels have demonstrated success in controlled experimental settings, translating these findings into clinically relevant scenarios faces challenges. Issues such as long-term biocompatibility, integration with host tissues, and scalability are critical factors that must be addressed before clinical application [121]. For example, the integration of stem cell-based therapies, such as those utilizing dental pulp stem cells or Schwann cells encapsulated within hydrogels, has shown potential in bridging nerve gaps and promoting functional recovery. However, the long-term viability of these cells within the hydrogel scaffold and their sustained release remains unresolved concerns that require further investigation [81].

Technological innovations, including conductive hydrogels and stimuli-responsive systems, have greatly enhanced the versatility of these materials in nerve repair. As noted, conductive hydrogels were shown to support electrical signal transmission, which is crucial for restoring nerve function. However, achieving consistent and reproducible results across different injury models remains a challenge. Studies have demonstrated that these hydrogels can facilitate electrical stimulation and improve axonal regeneration, yet their application across various models and injury types has not been consistently successful [71]. Similarly, drug delivery and cell-encapsulating hydrogels offer sustained and localized therapeutic effects, but maintaining the stability and bioactivity of encapsulated molecules, such as neurotrophic factors or therapeutic drugs, continues to present significant hurdles in vivo [76].

The interplay between hydrogels and the immune response is another critical area that needs further exploration. While some hydrogels were designed to modulate inflammation and promote macrophage polarization toward a pro-regenerative phenotype, their effectiveness can be influenced by the host’s immune environment, particularly in systemic conditions like diabetes, where oxidative stress can inhibit regeneration [92]. For example, studies examining the role of magnesium-enriched hydrogels have highlighted the importance of modulating the inflammatory response to enhance tissue repair and functional recovery [87]. Further, age-related constraints may also need to be addressed, as the immune response can vary significantly between younger and older individuals. It is now well established that aging is associated with a decline in immune function, often referred to as “immunosenescence”, which can affect the body’s ability to respond to injuries and repair damaged tissues [122]. In older individuals, we see a decrease in the inflammatory response, with increased chronic low-grade inflammation and impaired immune cell function, which could also affect the regenerative potential of hydrogel-based therapies. For example, studies have shown that older subjects exhibit delayed macrophage polarization, which is crucial for tissue repair and regeneration, potentially reducing the effectiveness of hydrogels designed to modulate the immune environment. Addressing these immune-related challenges requires the integration of insights from immunology, biomaterials science, cell and molecular biology, and regenerative medicine to better understand the dynamic interactions between the host immune system and hydrogel materials [93].

Regulatory and ethical considerations add another level of complexity to the clinical translation of hydrogel-based therapies. The development and approval of these therapies must meet strict safety and efficacy standards, especially when incorporating cellular or genetic components, such as stem cells or genetically modified materials [94]. Moreover, the high development costs associated with advanced hydrogels, along with the need for specialized manufacturing processes and the scalability of production, pose significant economic barriers. These challenges are particularly pronounced in low-resource settings, where access to cutting-edge therapies may be limited. Collaborative efforts between academia, industry, and regulatory bodies are essential to streamline the approval process and ensure that hydrogel-based therapies can be accessible to all patients.

Future research should focus on the standardization of hydrogel formulations and experimental protocols to facilitate more robust comparisons across studies and optimize treatment outcomes. Additionally, advances in materials science, such as the integration of nanotechnology, smart materials, and biocompatible conductive polymers, hold great promise for expanding and enhancing the therapeutic repertoire and efficacy of hydrogels. Future innovations could enable more precise control over the release of bioactive molecules, improve the integration of cells with the host tissue, and could in turn promote more effective nerve regeneration. Finally, continued collaboration between academic and industry researchers, clinicians, and regulatory authorities is crucial for overcoming the technological, regulatory, ethical, and socioeconomic barriers that currently limit the widespread clinical application of hydrogel-based therapies for peripheral nerve regeneration.

## 7. Conclusions

This review highlights the substantial promise of hydrogel-based technologies in advancing peripheral nerve repair. By recreating key aspects of the native ECM, hydrogels effectively support cell adhesion, proliferation, and differentiation while also enabling the controlled release of bioactive molecules. These advantages offer a clear improvement over traditional strategies, particularly when considering the versatility of hydrogels to be functionalized for highly personalized therapeutic applications. Moreover, the minimally invasive delivery of injectable hydrogels underscores their clinical value by reducing both patient recovery time and surgical risks. Moving forward, the principal aims in this arena, include refining hydrogel formulations for maximal therapeutic benefit, devising standardized protocols to accelerate clinical translation, and performing long-term efficacy and safety assessments to facilitate clinical adoption. By synthesizing current achievements and outlining potential future directions, this review could provide a valuable resource for researchers, clinicians, and biomaterials scientists seeking to study the potential use of hydrogels in peripheral nerve repair.

## Figures and Tables

**Table 1:** Overview of hydrogel scaffold components for guiding axonal growth.

Active Components/Features	Hydrogel Materials	Loaded Drugs/Active Components	Study Models	Effects on Recovery	Ref.
Polycaprolactone, chitosan, hydroxyapatite	Polycaprolactone/chitosan–hydroxyapatite	None	Preclinical, in vitro studies	Enhanced nerve guidance, axonal regrowth, improved implant stability and mechanical properties	[87]
RAD peptides (IKV, RGI, IKV/RGI)	RAD/IKV, RAD/RGI, RAD/IKV/RGI	None	Animal study (rat model)	Enhanced Schwann cell adhesion, myelination, neurotrophin secretion, axonal regeneration, functional recovery	[88]
Peptide nanofiber scaffold with chitosan conduits	Self-assembling peptide nanofiber scaffold (RAD/KLT, RAD/IKVAV, RAD/KLT/IKVAV), chitosan conduits	None	In vitro (Schwann cell assays), in vivo (rat model)	Improved nerve healing, remyelination, axonal regeneration, better muscle innervation and weight.	[64]
Decellularized ECM derived from bone, liver, and small-intestinal submucosa	Decellularized extracellular matrix (dECM) hydrogels	None	In vitro (DRG neurite extension), in vivo (rat sciatic nerve injury model)	Improved structural integrity, enhanced Schwann cell alignment, promoted vascularization	[73]
Visible light-crosslinked gelatin	PLCL with visible light-crosslinked gelatin hydrogel	None	In vivo (rat sciatic nerve defect model)	Demonstrated axonal regeneration, remyelination, functional recovery	[76]
Decellularized porcine nerve-derived ECM	Decellularized porcine nerve-derived ECM hydrogels	None	Preclinical (rat model, 24-week study)	Improved axon count, electrophysiological recovery, functional gait recovery comparable to autografts.	[65]
VEGF, NGF	Gelatin methacrylate (GM) hydrogels combined with chitin conduits	VEGF and NGF	In vitro (cell proliferation, migration, apoptosis), in vivo (rat model)	Enhanced nerve regeneration, improved nerve conduction velocity, reduced muscle atrophy, functional recovery	[63]
Wnt5a	Wnt5a-loaded fibrin hydrogels	Wnt5a	In vitro (Schwann cell assays), in vivo (rat model)	Improved axonal growth, myelination, Schwann cell proliferation, VEGF and NGF secretion	[62]
Multidomain peptides (K2, K2-IIKDI, K2-IKVAV)	Multidomain peptide (MDP) hydrogels	None	In vitro (neurite outgrowth), in vivo (rat crush injury model)	Accelerated functional recovery, enhanced macrophage recruitment, faster axonal regeneration	[67]
Graphitic carbon nitride (g-C3N4), reduced graphene oxide (rGO)	Graphitic carbon nitride (g-C3N4) and reduced graphene oxide (rGO)-based hydrogels	None	In vitro (PC12 cell studies), in vivo (nerve guidance conduit models)	Enhanced neurite extension, anisotropic guidance, optimal mechanical properties, biocompatibility	[89]
Gelatin methacryloyl (GelMA), silk fibroin methacrylate (SF-MA)	Gelatin methacryloyl (GelMA) and silk fibroin methacrylate (SF-MA) hydrogels	None	In vitro (Schwann cell assays), in vivo (rat sciatic nerve defect model)	Promoted axonal elongation, enhanced Schwann cell adhesion, proliferation, migration, functional recovery	[90]
Schwann cells encapsulated in chitosan–collagen hydrogel	Schwann cell-encapsulated chitosan–collagen hydrogel nerve conduit	None	In vitro (SC survival), in vivo (rat sciatic nerve defect model)	Promoted axonal regrowth, enhanced remyelination, better motor functional recovery	[91]
Decellularized porcine vagus nerve tissue	Peripheral nerve-derived hydrogels	None	In vitro (Schwann cell and sensory neuron culture), in vivo (rat sciatic nerve gap model)	Promoted axonal regeneration, supported Schwann cell viability, enhanced neurite outgrowth	[92]
Decellularized nerve matrix	Decellularized nerve matrix hydrogel-coated nanofibrous scaffolds	None	In vitro (Schwann cell migration, axonal outgrowth), in vivo (rat sciatic nerve defect model)	Promoted axonal growth, Schwann cell migration, enhanced myelination, functional recovery similar to autografts	[74]

**Table 2:** Barrier hydrogel scaffolds for reducing scar tissue.

Active Components/Features	Hydrogel Materials	Loaded Drugs/Active Components	Study Models	Effects on Recovery	Ref.
Dopamine–isothiocyanate and decellularized nerve matrix	Dual-network nerve-adhesive (DNNA)	None	In vitro (SC proliferation, axonal outgrowth), in vivo (rat sciatic nerve transection model)	Enhanced axonal outgrowth, reduced inflammation and fibrosis, improved motor and sensory recovery	[94]
M2-derived cytokines and extracellular vesicles (EVs)	Bionic peptide hydrogel scaffolds	M2-derived cytokines and EVs	In vitro (SC and macrophage behavior), in vivo (rat sciatic nerve gap model)	Promoted M2 macrophage polarization, enhanced SC migration, axonal regeneration, functional recovery	[93]
Reactive oxygen species (ROS)-triggered H2S release	Thermosensitive poly(amino acid) hydrogel (mPEG-PA-PP)	H2S	In vitro (SC, macrophage, endothelial cells), in vivo (rat sciatic nerve transection model)	Enhanced nerve regeneration, reduced oxidative stress and inflammation, promoted angiogenesis, functional recovery	[95]
Polydopamine nanoparticles (PDA NPs)	Polydopamine nanoparticles@hyaluronic acid methacryloyl (PDA NPs@HAMA) hydrogel	None	In vivo (rat sciatic nerve adhesion model)	Reduced nerve adhesion, improved motor nerve conduction, reduced inflammatory response, better nerve functionality	[96]

**Table 3:** Conductive hydrogel components for enhancing electrical function.

Active Components/Features	Hydrogel Materials	Loaded Drugs/Active Compounds	Study Models	Effects on Recovery	Ref.
Conductive polymer	Electroconductive hydrogel	None	In vivo (diabetic sciatic nerve injury model)	Promoted axonal regeneration, remyelination, improved motor function, reduced muscle atrophy	[77]
Polypyrrole (PPy), tannic acid (TA), ropivacaine microspheres	Electroconductive hydrogel with PPy and TA	Ropivacaine microspheres	In vitro (SC, PC12 cell assays), in vivo	Enhanced axonal regeneration, myelination, reduced muscle atrophy, long-acting analgesia, functional recovery	[98]
Conductive electrolytic material	Conductive electrolytic hydrogel integrated in a nerve cuff	None	In vivo (rat sciatic nerve model)	Effective neuromodulation, reversible nerve block, comparable stimulation and recording to traditional electrodes	[99]
Gold nanoparticles	Alginate/poly-acrylamide hydrogel	Gold nanoparticles	Rodent and porcine nerve injury models	Enhanced motor/sensory recovery, axonogenesis, muscle mass preservation, atraumatic electrode removal	[100]
Collagen, alginate, GelMA, PEGDA	Multi-component hydrogel	None	In vitro (PC12 cell differentiation studies)	Enhanced PC12 cell differentiation and neurite outgrowth, dependent on hydrogel type and stimulation	[101]
Fatigue-resistant nanocrystals	Polyvinyl alcohol (PVA) nanocrystalline hydrogel optical fibers	None	In vivo (mouse models, optogenetics)	Enabled stable optogenetic nerve modulation, reduced pain hypersensitivity, facilitated motor recovery	[102]

## Data Availability

Not applicable.
